# Potential Systematic Interception Errors are Avoided When Tracking the Target with One’s Eyes

**DOI:** 10.1038/s41598-017-11200-5

**Published:** 2017-09-07

**Authors:** Cristina de la Malla, Jeroen B. J. Smeets, Eli Brenner

**Affiliations:** Department of Human Movement Sciences, Vrije Universiteit Amsterdam, NL – 1081BT, Amsterdam, The Netherlands

## Abstract

Directing our gaze towards a moving target has two known advantages for judging its trajectory: the spatial resolution with which the target is seen is maximized, and signals related to the eyes’ movements are combined with retinal cues to better judge the target’s motion. We here explore whether tracking a target with one’s eyes also prevents factors that are known to give rise to systematic errors in judging retinal speeds from resulting in systematic errors in interception. Subjects intercepted white or patterned disks that moved from left to right across a large screen at various constant velocities while either visually tracking the target or fixating the position at which they were required to intercept the target. We biased retinal motion perception by moving the pattern within the patterned targets. This manipulation led to large systematic errors in interception when subjects were fixating, but not when they were tracking the target. The reduction in the errors did not depend on how smoothly the eyes were tracking the target shortly before intercepting it. We propose that tracking targets with one’s eyes when one wants to intercept them makes one less susceptible to biases in judging their motion.

## Introduction

In daily life people usually direct their gaze towards objects with which they want to interact^1–4^. Fixating a position some distance away from a target that we are aiming for with our hand leads to a clear drop in the accuracy of various arm movements^5–11^, which suggests that looking at a target is beneficial for successfully performing an arm movement towards it. We also track moving objects with our eyes when we intend to hit or catch them^12–15^. Tracking targets increases the precision with which we can hit them^13^ and the precision with which we can anticipate how they will continue to move when they disappear from view^16, 17^. Nevertheless, continuous smooth and accurate pursuit of the target (i.e. constantly looking exactly at the target) does not seem to be necessary to succeed in interception tasks that require high precision, such as hitting a ball^18–20^.

In order to intercept a target one must consider both its velocity and its position. When judging how a target moves from how its image moves across the retina (for instance because one is fixating a static structure instead of tracking the target), many factors are known to influence the target’s apparent position and velocity, such as its contrast, luminance, spatial frequency and any embedded motion^21–34^. Such influences can disappear when tracking the target;^35^ for reviews see refs 36, 37). Since systematic errors in the perception of position or velocity lead to equivalent errors in interception^34^, not tracking the target will allow such factors to give rise to systematic errors in interception.

Reducing the effect of different factors on the target’s perceived movement is only one of the reasons why tracking a target with one’s eyes when trying to intercept it might be useful. The most obvious reason to track the target with one’s eyes is that looking at the target maximizes the spatial resolution near the target. However, interception does not appear to suffer much from a decrease in spatial resolution^38–40^. Another possible reason is that tracking the moving target with one’s gaze changes the way in which the object’s motion is judged: instead of combining fast retinal slip of the (small) target image with signals related to the absence of movement of the eyes, modest amounts of retinal slip of the target’s image are combined with information about the speed of rotation of the eyes. To estimate the rotation of the eyes, extra-retinal sources of information and the fast retinal slip of images of (large) surrounding stationary structures can be combined^41^. This combination of two sources of information might improve the precision with which the target’s motion is judged, and thus reduce reliance on prior expectations^42^. Another potential advantage of relying on information about the eyes’ movements to judge the target’s speed is that during smooth pursuit, retinal slip of the target’s image provides direct feedback about errors in keeping the eyes on the target, and thus about one’s judgments of the target’s speed. Such feedback can obviously be used to update one’s judgment. In this study we focus on the first reason for tracking the target with one’s eyes. We explore whether tracking a target helps prevent a factor that is known to give rise to systematic errors in judging retinal speeds from resulting in systematic errors in interception.

To test this hypothesis we conducted two experiments in which subjects had to intercept moving targets. The targets were either uniform or patterned disks. Subjects had to either track the target with their gaze until they tapped on it, or fixate a certain position, and tap on the target when it reached that position. We biased motion perception by adding local motion within the patterned targets^34^. Such motion was either in the same direction as, or in the opposite direction than, the direction in which the target was moving. Moving the pattern within the target gave rise to systematic errors in interception when subjects were fixating: subjects tapped several centimetres ahead of the target when the pattern moved in the same direction as the target, and several centimetres behind the target when the pattern moved in the opposite direction than the target. Tracking the targets with their eyes dramatically reduced the errors that subjects made.

## Methods

### Subjects

Ten subjects took part in Experiment 1 and fourteen subjects took part in Experiment 2. They were all naïve with respect to the purpose of the study. After applying the inclusion criteria (see *Data analysis*) we were left with nine subjects’ data for each experiment. Four of these subjects took part in both experiments. All subjects reported being right handed. They all had normal or corrected-to-normal vision. None had evident motor abnormalities. All gave written informed consent. The study was part of a program that was approved by the local ethical committee of the Faculty of Behavioural and Movement Sciences at VU University. The experiments were carried out in accordance with the approved guidelines.

### Apparatus and calibration

The experiments were conducted in a normally illuminated room. Subjects stood in front of a large back-projection screen (Techplex 150, acrylic rear projection screen; width: 1.25 m, height: 1.00 m; tilted backwards by 30°) onto which the stimuli were projected (InFocus DepthQ Stereoscopic Projector; resolution: 800 by 600 pixels; screen refresh rate: 120 Hz). The setup gave subjects a clear view of the stimuli as well as of their arm, hand and finger. Subjects intercepted the projected targets by tapping on the screen. They were not restrained in any way. An infrared camera (Optotrak 3020, Northern Digital) that was positioned at about shoulder height to the left of the screen measured the position of a marker attached to the nail of the subjects’ index finger at 250 Hz. A head-mounted eye-tracking system (Eyelink II, SR Research) measured the movements of both eyes at 500 Hz. We used a biteboard with a dental imprint and three infrared markers attached to it to also register the head movements with the Optotrak.

We first calibrated a pointer, consisting of a rod with a tapered end and three infrared markers attached to it, by placing an additional marker at the tip of the rod and determining the position of this marker (and therefore of the tip) with respect to the three markers attached to the pointer. We then used the pointer to calibrate the screen (in terms of Optotrak coordinates).

For each subject we then calibrated the biteboard to determine the positions of the eyes with respect to the three markers attached to it. To do so, we placed the previously calibrated pointer on a tripod and displayed a small white target on the screen. Subjects were asked to align the tip of the rod with the white dot while only looking with one eye. They could move any way they wanted to do so. When they considered the tip of the rod and the dot on the screen to be properly aligned they pressed the button of a computer mouse that they were holding in their hand. Once they did so, a new dot appeared at a different position on the screen (unless they had moved their head by more than 1 mm during the last 300 ms before pressing the button, in which case they had to try again). They aligned the tip of the rod with the dot 20 times for the left eye and 20 times for the right eye. Each measurement provided us with a line with respect to the biteboard: the line connecting the known positions of the tip of the rod and of the target on the screen. These lines should all pass through the eye in question. The position that minimized the combined distances to the lines was considered to be the position of the eye (all determined with respect to the biteboard), so that we could determine the positions of the two eyes by measuring the position of the biteboard from then on.

Once the biteboard had been calibrated, we could calibrate the eye movement recordings. To do so, we presented a dot at the centre of the screen, and asked subjects to move their heads while maintaining fixation on the dot for 30 s. By combining the position of the dot relative to the head from the Optotrak data with the coordinates of the pupil with respect to the head from the Eyelink data, we could relate the Eyelink coordinates to gaze angles. We verified the calibration by asking subjects to fixate indicated positions on the screen and rendering dots at the positions at which we considered the subjects to be looking with their left and right eyes. If subjects reported that the dots were at the positions they were looking, the calibration was considered correct. If not, the calibration was repeated following the same procedure.

The final step in the calibration was to measure the position of the marker on the fingertip when the subject placed the fingertip at four indicated positions on the screen. This was used to relate the position of the fingertip marker to where the subject perceived his finger relative to the projected images, automatically correcting for the fact that the marker was attached to the nail rather than to the tip of the finger.

We synchronized the Optotrak recordings with the images projected on the screen by flashing a disk in the upper left corner of the screen whenever a new target appeared. This flash activated a sensor that was directed towards that part of the screen, which briefly inactivated an additional Optotrak marker attached to the side of the screen. Detecting this inactivation provided information (to within 4 ms) about when the target appeared relative to the movement data. Using this sensor we also determined that the latency with which we could adjust the images to events extracted from the online Optotrak data was 24 ms. We used the actual timing, to within 4 ms, for the analysis as well as for the feedback during the trials. Subjects did not notice that the target moved on for about 24 ms before feedback was provided, presumably partly because their own finger occluded the target and partly through backward masking.

### Stimulus and procedure

**Figure 1:**
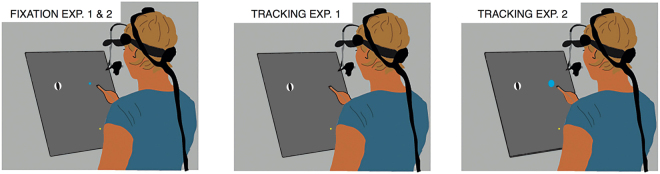
Schematic representation of the task. Subjects had to intercept a target (a patterned disk, as shown, or a solid one) by tapping on it. In different trials they either had to fixate the position at which they had to tap the target (Fixation condition, blue dot in left panel), or to track the target with their eyes (Tracking condition) in which case they could either tap the target wherever they wanted (middle panel) or had to tap on it within a certain area (blue region in right panel).

The general procedure for the two experiments is shown in Fig. 1. Subjects started each trial by placing their index finger at an indicated starting point. The starting point was a 1.4 cm diameter yellow disk that was 10 cm to the right of and 15 cm below the screen centre. Between 500 and 800 ms after the finger was placed at the starting point, a target appeared on the left side of the screen, 25 cm above the starting point. It was a 7 cm diameter disk that moved from left to right across the screen. Subjects had to try to intercept the target by tapping on it. Taps were detected on-line. A tap was considered to have occurred if the deceleration of the movement orthogonal to the screen was at least 100 m/s^2^ while the finger was less than 5 mm above the screen and within 10 cm of the target’s path. Whenever they wanted, subjects could rest between trials by not placing their finger at the starting point.

There were three types of targets: *Backwards*, *Neutral* and *Forward*. *Neutral* targets were white disks that moved with a constant velocity of 50, 60 or 70 cm/s. We used various slightly different velocities in order to force subjects to judge the velocity. The *Backwards* and *Forward* targets were disks with a pattern that moved within the targets. These targets moved with a constant velocity of either 55 or 65 cm/s. The pattern within the disks consisted of a simulation of vertical segments, like on a beach ball, rotating around the point at which all eight segments meet, projected in orthographic perspective onto the screen. The segments’ colours alternated between black and white. The *Forward* targets can be considered to be simulations of a rolling ball seen from above, so the pattern within the target moved twice as fast as the target itself (in the same direction) when it was at the centre of the target. The speed of the pattern decreased with the distance from the centre as it does for a real rolling ball. The pattern within the *Backwards* targets moved in the same way but in the opposite direction (as in a ball with backspin). Thus, the pattern within the *Backwards* targets was static when it was at the centre of the target and its speed increased with the distance from the centre to reach the same speed as the target itself at the edges of the target.

Both experiments consisted of randomly interleaved trials in which subjects either had to fixate the position at which the target was to be tapped (Fixation condition; left panel in Fig. 1) or to track the target with their eyes (Tracking condition; middle and right panels in Fig. 1). To start each trial, subjects had to place their finger at the starting position. Once they did that, the target appeared, moving from left to right across the screen. The position at which the target appeared was different for each target velocity (see Table 1). In the trials of the Fixation condition, the fixation position was indicated by a blue 1.6 cm diameter fixation dot. We varied the time that it took for the targets to reach the fixation dot. This could be 650, 700 or 750 ms. Consequently, the fixation dot’s position was determined by the combination of the target’s velocity and the time needed for the target to reach the tapping position. Subjects were required to tap on the target when it was superimposed with the fixation dot. In Experiment 1, if no fixation dot appeared when subjects placed their finger at the starting position, subjects had to track the target with their eyes. In this Tracking condition, the positions at which the targets appeared and their velocities were the same as in the Fixation condition, but subjects could tap the target anywhere they wanted along its path (middle panel in Fig. 1).

**Table 1:** Details of the target movements for the Fixation conditions of both experiments. In the Tracking condition of Experiment 1 there was no fixation point so the time to tapping position and location of fixation point are irrelevant, but the other details are the same. In the Tracking condition of Experiment 2, it was the centre of the 6 cm tapping area, rather than that of the fixation point, that was at the mentioned location.

Type of target	Velocity (cm/s)	Starting position (cm)	Time to tapping position (ms)	Location of fixation point (cm)	Trials
Backwards	55	−28.5	650	7.2	5
Backwards	55	−28.5	700	10	5
Backwards	55	−28.5	750	12.8	5
Backwards	65	−35.5	650	6.8	5
Backwards	65	−35.5	700	10	5
Backwards	65	−35.5	750	13.2	5
Neutral	50	−25	650	7.5	5
Neutral	50	−25	700	10	5
Neutral	50	−25	750	12.5	5
Neutral	60	−32	650	7	5
Neutral	60	−32	700	10	5
Neutral	60	−32	750	13	5
Neutral	70	−39	650	6.5	5
Neutral	70	−39	700	10	5
Neutral	70	−39	750	13.5	5
Forward	55	−28.5	650	7.2	5
Forward	55	−28.5	700	10	5
Forward	55	−28.5	750	12.8	5
Forward	65	−35.5	650	6.8	5
Forward	65	−35.5	700	10	5
Forward	65	−35.5	750	13.2	5

To make sure that any differences between tapping errors in the Fixation and Tracking conditions of Experiment 1 were not due to subjects benefiting from the possibility of tapping whenever and wherever they wanted in the Tracking condition^43^, we conducted a second experiment in which we also presented a tapping area in the Tracking condition. In Experiment 2 everything was exactly the same as in Experiment 1 except that in the Tracking condition, after subjects placed their finger at the starting position, a 6 cm diameter blue disk (tapping area) was shown at the position at which the fixation dot would have appeared in the equivalent trial of the Fixation condition (i.e. for the same combination of target speed and time to reach the tapping position; right panel in Fig. 1 and Table 1). This tapping area was much larger than the fixation point in the Fixation condition, because otherwise subjects would not be able to pursue the targets but would look at the tapping area instead^13^.

Feedback about the tapping performance was provided on each trial. If subjects hit the target, the target stopped moving. If they hit it at the right place, a sound indicated that the trial was successful. For the Fixation conditions the ‘right place’ was within the fixation dot. For the Tracking condition of Experiment 2 the ‘right place’ was within the tapping area. For the Tracking condition of Experiment 1 any place was right, so a sound was presented whenever the target was hit. If the tap missed the target, the latter deflected away from the finger at 1 m/s, remaining visible for 500 ms.

In each experiment there were 30 trials for each of the seven combinations of type of target and velocity, 15 in which subjects had to fixate and 15 in which subjects had to track (see Table 1). On each trial, a combination of type of target, time to tapping position, velocity of the target, and Fixation or Tracking condition, was selected at random from the remaining trials that were to be done. In addition to these 210 trials, each experiment started with 20 practice trials to familiarise the subjects with the task (those trials were not included in the analyses). In the practice trials the targets were *Neutral*, the velocity at which they moved was 60 cm/s, the starting position was 32 cm to the left of the screen centre, and if there was a fixation dot or tapping area it appeared 10 cm to the right of the screen centre (so it took 700 ms for the centre of the target to reach the tapping position). Ten of the practice trials were similar to trials of the Fixation condition and the other ten were similar to those of the Tracking condition. The total of 230 trials took about 20 minutes to complete for each experiment.

### Data analysis

All analyses were performed with R Statistical Software^44^. For each trial we determined the tapping error: the horizontal distance between the position at which subjects tapped the screen and the position of the centre of the target at the moment of the tap. Trials in which the tapping error was more than three standard deviations from the mean for each subject, velocity, type of target (*Backwards*, *Neutral*, *Forward*) and condition (Fixation or Tracking) were removed from the analysis, to be sure to remove any trials in which the subject was not trying to tap on the target or in which the tap was not detected correctly. Systematic tapping errors were obtained for each subject, type of target and condition by averaging across trials with the same velocity (ignoring the time that it took for the target to reach the tapping position) and then across velocities. We also measured the difference between the position of the tap and the centre of the fixation point (in the Fixation condition) or of the tapping area (in the Tracking condition in Experiment 2) to make sure that subjects were performing the task as required. Subjects did indeed conform to these requirements, but the requirement of tapping the targets within a certain area made it more difficult to pursue the target with the eyes. We therefore had to exclude many trials and subjects in Experiment 2 (as will be explained below).

We subjected the systematic tapping errors to repeated measures ANOVAs to test whether there was an interaction between the effect of the type of target (*Backwards*, *Neutral* or *Forward*) and the condition (Fixation or Tracking). We anticipated that when subjects track targets with their gaze (Tracking condition), the systematic differences between the errors in intercepting the different target types will be smaller than when the target moves across the retina (Fixation condition), so there should be a significant interaction between type of target and condition.

Besides evaluating the tapping errors, we also examined the subjects’ eye movements. We analysed the eye movements during the last 400 ms of each trial. By that time, subjects had shifted their gaze from the starting position of the index finger to the target or fixation point. In trials of the Tracking condition they had sometimes also briefly looked at the region in which they tapped, presumably to check whether a small blue dot had appeared (in which case they would have had to fixate the dot). We determined where subjects were looking on the basis of the average measurements of the two eyes. We expected tracking the target during the period between 300 and 100 ms before the tap to be critical. Sensory information acquired 100 ms before the tap or later cannot influence the ongoing movement due to sensorimotor delays^43, 45, 46^. Earlier than about 300 ms before the tap we anticipated that subjects might not always have had enough time to direct their gaze at the target and track it reliably.

**Figure 2:**
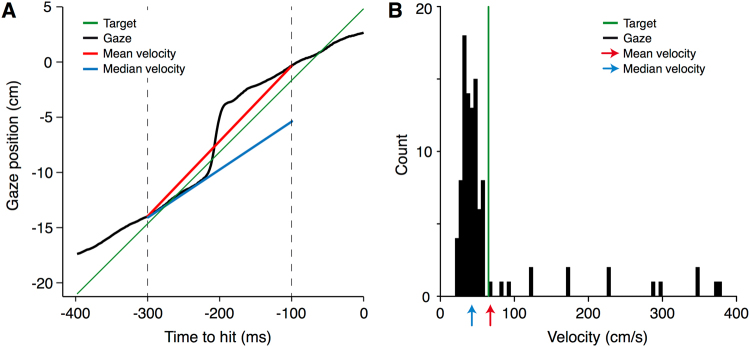
Method used to calculate the pursuit gain. (**A**) The gaze position on one trial, as a function of the remaining time until the moment of the tap (black curve). The two vertical dashed lines indicate the borders of the period that we use to evaluate the pursuit gain. (**B**) Histogram of the 100 samples of gaze velocity during this period. Note that the median velocity (blue line and arrow) is a reasonable estimate of the smooth component of tracking, despite the presence of the saccade. The extent to which the mean velocity is influenced by the saccade is also shown (red line and arrow). The target’s position and velocity are shown in green.

As we are interested in the effect of eye movements on interception behaviour, we only analysed trials in which subjects did follow the eye movement instructions (Fixate or Track). As a measure of whether participants followed the instruction about tracking the target during this critical period of time, we divided the horizontal change in gaze position by the target’s displacement during this period (Fig. 2). We removed trials in which the error in tracking the target during the critical period was more than 30%. For trials in which subjects should have tracked the target, we removed trials in which the change in gaze position was less than 70% or more than 130% of the target displacement. For trials in which subjects should have fixated, we removed trials in which the change in gaze position was more than 30% of the target displacement, either in the direction of target motion or in the opposite direction. Subjects for whom more than 60% of the trials in the Tracking condition were removed were altogether excluded from the analyses (one subject in Experiment 1 and 5 subjects in Experiment 2). For the remaining subjects, 9.8% of the trials were removed in Experiment 1 and 25% of the trials were removed in Experiment 2.

In the Tracking condition participants often followed the target with a combination of smooth pursuit and saccades. Figure 2A shows an example of the changes in gaze in one of the included trials containing a saccade. To calculate the pursuit gain, we first determined the horizontal gaze velocity at each time point by dividing the difference between the horizontal positions of gaze 10 ms after and 10 ms before the moment in question by the 20 ms time difference between them. We determined the acceleration by dividing the difference between the horizontal velocities 10 ms after and 10 ms before the moment in question by the 20 ms time difference between them. We identified saccades by looking for velocity peaks that differed by more than two times the target’s velocity from the target velocity itself. Once such a peak was detected, we found the beginning of the saccade by localizing the moment at which the acceleration first reached a threshold of 5 cm/s^2^ (or of −5cm/s^2^ for the backwards saccades). The end of the saccade was defined as the moment at which the acceleration reached its minimum (or maximum for backwards saccades), after the peak velocity had been detected. This slightly underestimates the saccade duration, but it works reasonably well even when gaze velocity is adjusted after the saccade. We distinguished between saccades towards the tapping position and all other saccades (catch-up saccades and backward saccades). Saccades that ended within 6 cm of where subjects were looking at the time of the tap were considered saccades towards the tapping position.

As our measure of pursuit gain, we determined the median gaze velocity during the relevant 200 ms of each trial (indicated by the blue line in Fig. 2A). If a saccade had been made towards the tapping position, only the period before the saccade was considered when calculating the median velocity. If there was less than 100 ms before the onset of such a saccade (i.e. if the saccade started more than 200 ms before the tap), we considered the last 100 ms before the saccade (from up to 400 ms before the tap). To express this as a gain, we divided the median velocity of the change in gaze by the target’s velocity. Figure 2B shows a histogram of the gaze velocities during all 2 ms intervals of the period of interest for this example trial. This figure illustrates that the median velocity (blue arrow), which is determined by the *predominant* velocity at which the target was tracked rather than by the mean velocity (red arrow), corresponds well with the smooth pursuit component of tracking. Since using the median is simple, and circumvents having to pick an arbitrary threshold for saccade amplitude, we used the median to calculate the gain. We determined the mean pursuit gain for each type of trial (*Backwards*, *Neutral*, *Forward*). If we find that tracking the target decreases the influence of target type on the tapping errors, failing to properly track the target might be responsible for residual errors in the Tracking condition. If so, we might be able to explain systematic differences in tapping errors for the different types of trials by differences between the eye movements. We therefore examined whether there was any relationship between the tapping errors and the pursuit gain or the moment at which pursuit of the target ended.

We also made heat maps of subjects’ gaze to provide an overview of the participants’ eye-movement strategies. We corrected for shifts due to the eye-tracker slipping after the calibration by assuming that by 400 ms before the tap subjects were always looking at the fixation point during Fixation trials. We subtracted the measured fixation error at that moment from all gaze positions on that trial, as well as from all gaze positions on any subsequent Tracking trials (until the next Fixation trial).

## Results

### Tapping behaviour

**Figure 3:**
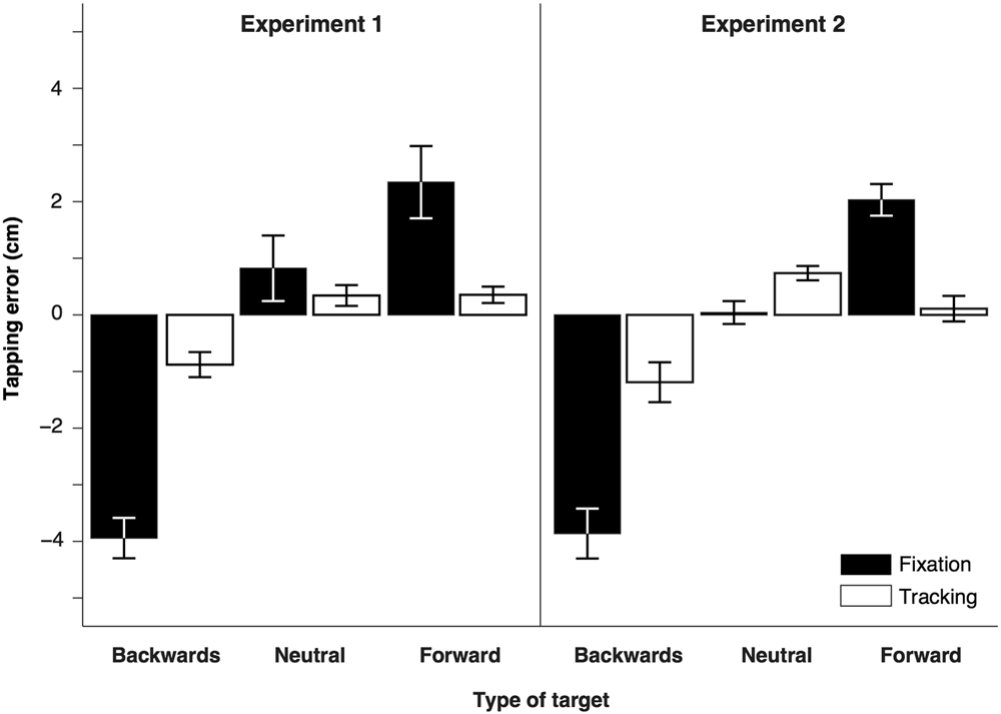
Mean horizontal distance between the centres of the finger and of the target for the three types of target (*Backwards*, *Neutral* and *Forward*) in both experiments. Positive values indicate tapping ahead of the target. Error bars are the standard error of the mean across subjects.

Figure 3 shows the systematic tapping errors for Experiment 1 (left) and 2 (right), averaged across subjects and velocities. Positive values indicate that subjects tapped ahead of the target. In the Fixation condition (black bars), motion within a moving target clearly influenced attempts to intercept it. Subjects tapped several cm behind the *Backwards* targets (in accordance with such targets appearing to move more slowly than they actually were) and several cm ahead of the *Forward* ones (in accordance with such targets appearing to move faster than they actually were). In the Tracking condition (white bars), the tapping errors were much reduced. When tracking the target with their eyes, the systematic errors for the *Backwards* and *Forward* targets were hardly larger than when the target was *Neutral*. Consequently, for both experiments, repeated measures ANOVAs on the systematic tapping errors revealed significant interactions between the type of target and the visual condition (Experiment 1: F(2,16) = 84, p < 0.0001; Experiment 2: F(2,16) = 50, p < 0.0001). The main effects of the type of target were also significant (Experiment 1: F(2,16) = 114, p < 0.0001; Experiment 2: F(2,16) = 79, p < 0.0001).

In the Tracking condition of Experiment 1, subjects were free to tap the target wherever they wanted, as long as they hit it. In all the other conditions they were required to tap the targets at a specific position or within a limited range of positions. Subjects accurately tapped on the fixation point in the Fixation conditions. Their mean errors (and their mean standard deviations) were respectively −0.6 (0.7), −0.2 (0.5) and 0 (0.4) cm for the *Backwards*, *Neutral* and *Forward* targets of Experiment 1 and −0.3 (0.4), −0.2 (0.4) and−0.2 (0.4) cm for the same targets of Experiment 2. Subjects also tapped well within the tapping area in Experiment 2, with mean errors (and mean standard deviations) of 0 (0.7), 0.3 (0.7) and 0.3 (0.6) cm for the *Backwards*, *Neutral* and *Forward* targets respectively. The reported errors are with respect to the centre of the fixation point or tapping area.

### Eye movements

**Figure 4:**
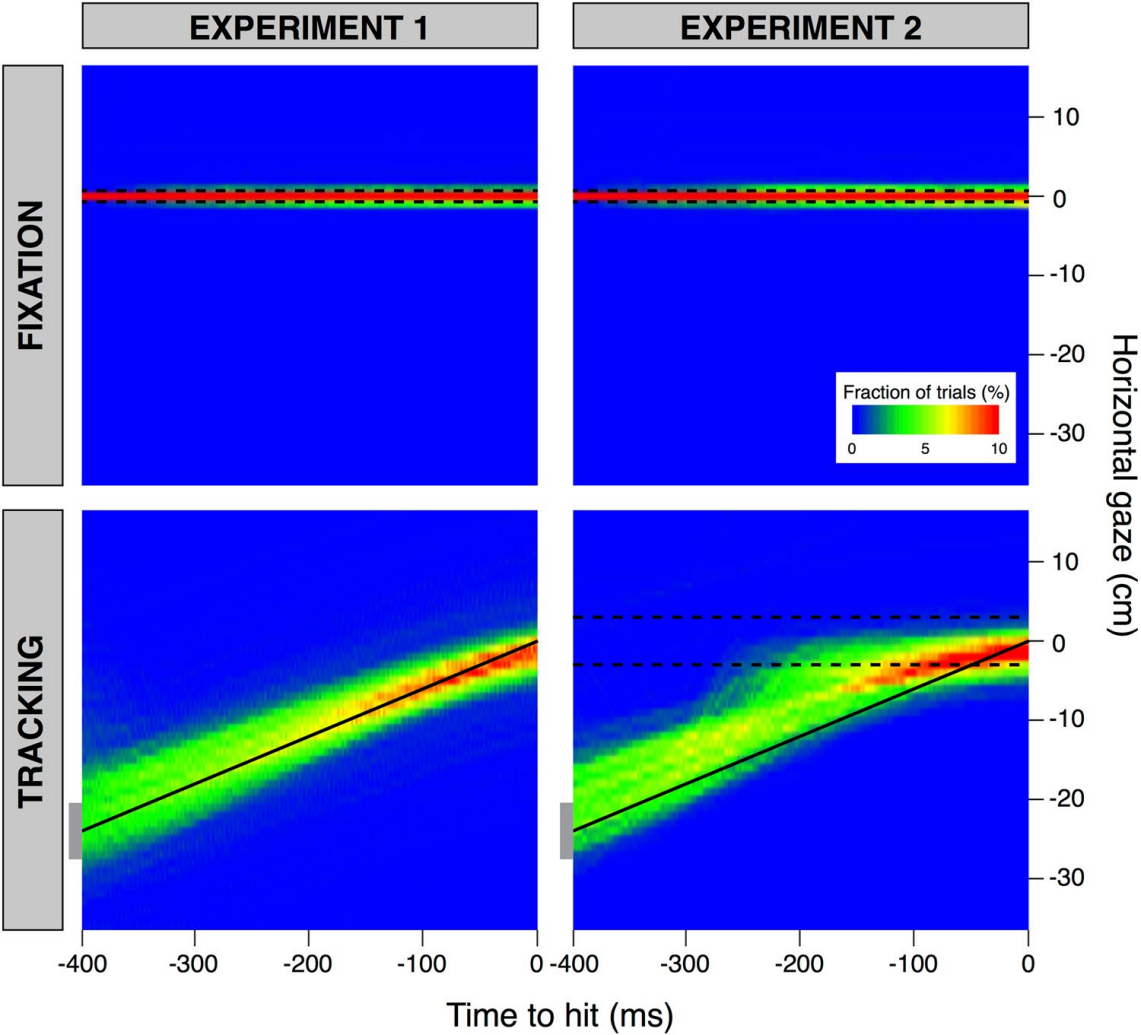
Heat maps of the horizontal distance of gaze from where the structure that subjects had to fixate was situated at the time of the tap, as a function of the remaining time until the moment of the tap. The structure that subjects had to fixate was the fixation point for the Fixation condition (upper panels) and the target for the Tracking condition (lower panels). Blue indicates that people seldom looked at the position in question at that time. Red indicates that they often did so. The dashed black lines indicate the maximal horizontal extent of the area within which subjects had to tap on the target (subjects were free to intercept the target wherever they liked in the Tracking condition of Experiment 1). The black line in the lower panels indicates the average position of the target at each moment (i.e. the position for a velocity of 60 cm/s). The grey rectangles at the left ends of these black lines indicate the size of the target.

**Figure 5:**
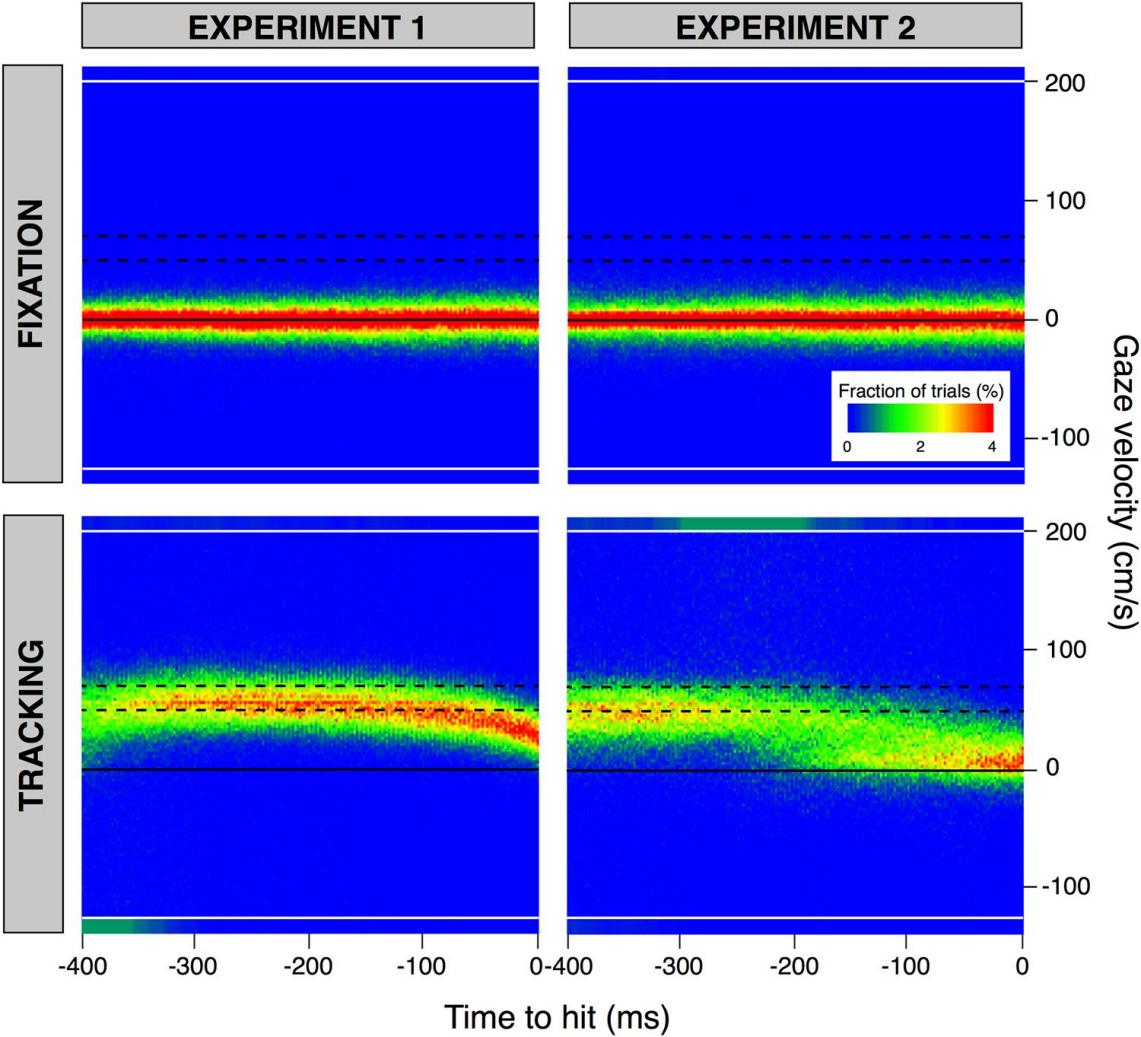
Heat maps of the horizontal gaze velocity as a function of the remaining time until the moment of the tap. The black line indicate a velocity of 0 cm/s. The dashed lines indicate the range of target velocities. The sections above and below the white lines represent the cumulative occurrences of values above 200 cm/s and below −125 cm/s, respectively. Such velocities are indicative of saccades. To give a graphical impression of the relative frequency of saccades, the fractions of trials within these sections are scaled to match the area in which these cumulative occurrences are represented: the height of each of these areas corresponds to that of five bins, so we divided the cumulative occurrences of values above 200 cm/s (and of values below −125 cm/s) by five.

The overall gaze behaviour during the trials is presented as heat maps in Figs 4 and 5. In Fig. 4 we show how far the horizontal component of gaze was from the position subjects had to fixate at the moment of the tap, as a function of the time with respect to the moment of the tap. The figure shows the horizontal distance from gaze to the fixation position for the trials in the Fixation condition (upper panels) and the horizontal distance from gaze to the position of the target at the time of the tap for the trials in the Tracking condition (lower panels). Separate columns show gaze behaviour for the two experiments. In the Fixation condition, the horizontal gaze should be close to zero throughout the trial, because subjects were asked to fixate the blue dot that indicated where they had to tap the target. Most values remain within the region indicated by the two dashed lines in the upper panels of Fig. 4, indicating that subjects maintained their gaze within the horizontal extent of the fixation point throughout the last 400 ms before the tap.

In the Tracking condition (lower panels of Fig. 4), gaze should be directed at the moving target, so on average the gaze should follow the black line that represents a target moving at 60 cm/s (the average target velocity). In Experiment 1, subjects’ gaze was slightly ahead of the target but tracked it quite accurately. Moreover, subjects tracked the target quite well: the gains of the pursuit between 300 and 100 before the moment of the tap were 0.83 ± 0.02, 0.93 ± 0.02 and 0.93 ± 0.03 for the *Backwards*, *Neutral* and *Forward* targets, respectively (mean ± standard error across subjects). The gain was lower for the *Backwards* targets than for the other two kinds of targets. In Experiment 2, subjects tracked the target reasonably accurately until about 200 ms before the tap. Around that time they often made a saccade to near where they would tap the target (where the blue tapping area was visible). The pursuit gains were slightly lower than the ones in Experiment 1: 0.72 ± 0.03, 0.79 ± 0.03 and 0.77 ± 0.04 for the *Backwards*, *Neutral* and *Forward* targets (mean ± standard error across subjects). Again, the gain was lower for the *Backwards* targets than for the other two kinds of targets. Even before the saccades to the tapping position, the subjects’ gaze was often further ahead of the target in the Tracking condition of Experiment 2 than at the same time in Experiment 1, probably because subjects needed to monitor the position of the area within which they were expected to tap the target in Experiment 2.

Some aspects of the eye movements are easier to see in plots of the gaze velocity (Fig. 5). In the Fixation condition, gaze velocity was close to zero (black line in upper panels). In the Tracking condition of Experiment 1, subjects often made leftwards saccades about 400 ms before the tap (green region below the lower line in the lower left panel) and then moved their eyes almost as fast as the target (range of possible target velocities indicated by dashed lines), until shortly before the tap, when the gaze velocity declined. The initial saccades brought the eyes from the starting position (or the area at which the fixation position could appear) to the target. We do not see as many such saccades for Experiment 2 (lower right panel) because in that case the time of the tap was fixed: subjects had to wait for the target to be within the tapping area. In Experiment 1 subjects could hit the target at any time they wanted, and they usually did so sooner than in the corresponding condition of Experiment 2. In Experiment 2 we see quite consistent smooth pursuit until about 300 ms before the tap, with many rightward saccades between 300 and 200 ms before the tap (green region above the upper line). These are saccades towards the tapping area (see Fig. 4). After these saccades, gaze velocity was clearly lower than that of the target.

### Relating the tapping errors to the eye movements

**Figure 6:**
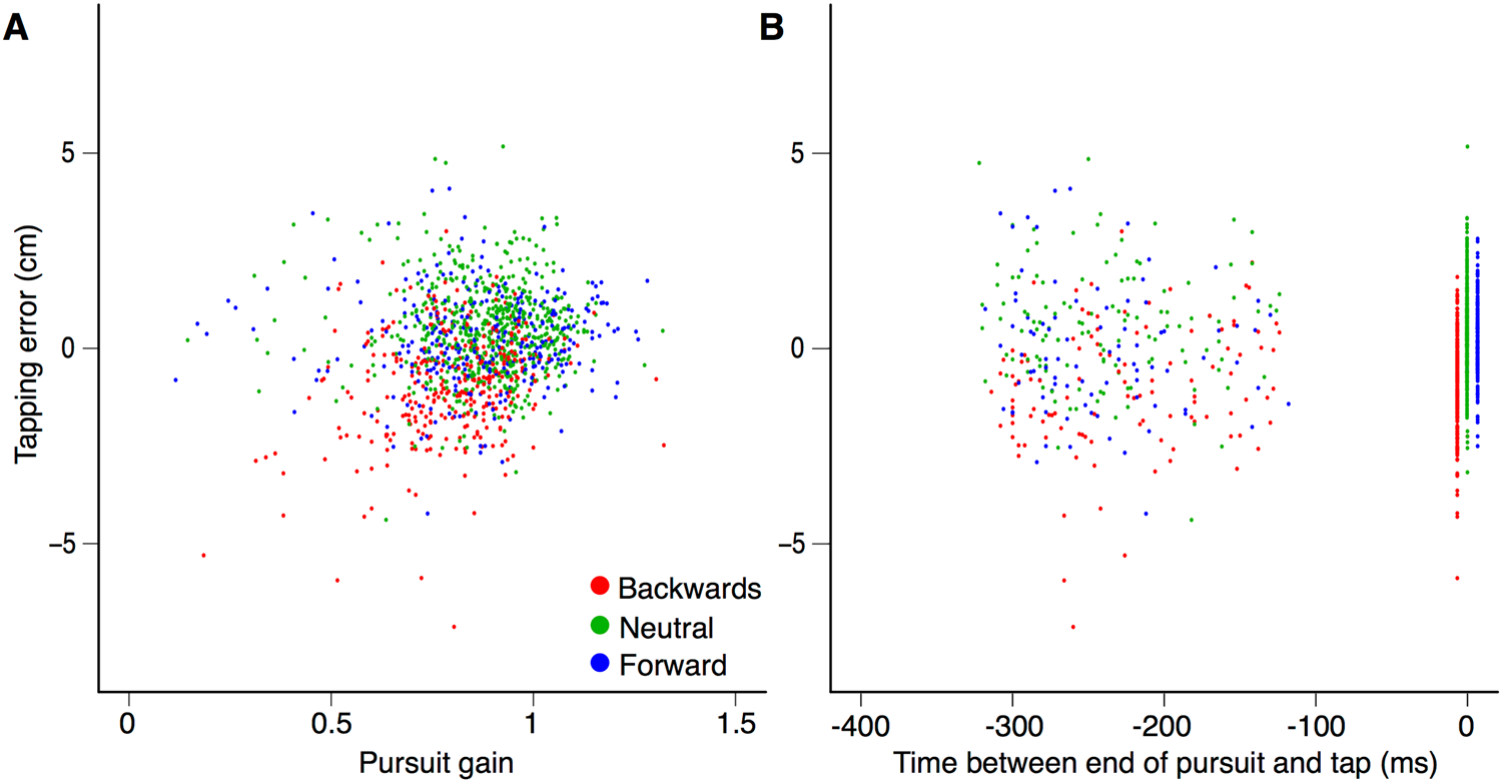
Tapping errors as a function of the pursuit gain (**A**) and of the time between when the pursuit ended and the tap (**B**). Trials of the Tracking condition of both experiments. Target types are colour-coded. The values near time 0 in panel B show the errors in trials in which there was no saccade to the tapping area. In this case, subjects continued to pursue the target, so that there was no end of pursuit before the tap. These values are therefore all considered to be zero, but the points in the figure have been shifted slightly for the Backwards and Forward conditions to ensure that the ranges remain visible for all types of targets.

In the Fixation conditions of both experiments, subjects followed the instruction and did not track the target (Figs 4 and 5). The considerable tapping error clearly depended on the type of target (Fig. 3). In the Tracking conditions, subjects also followed the instruction and tracked the target. The tapping errors were much smaller and more similar for the different types of target. The residual influence of the type of target on the tapping errors was similar in both experiments (Fig. 3), despite subjects tracking the target much less precisely in Experiment 2 than in Experiment 1 from 200 ms before the tap (Figs 4 and 5). This shows that subjects do not need to pursue a target smoothly until they tap on it for the action to be less susceptible to the motion within the target. It is enough to have tracked the target with one’s eyes for some time. Further evidence for this is shown in Fig. 6. Figure 6A shows that the error in tapping does not depend on the pursuit gain. The correlation coefficients between the tapping error and the pursuit gain are r = 0.26 (p < 0.01), r = −0.02 (p = 0.7) and r = 0.03 (p = 0.6) for the *Backwards*, *Neutral* and *Forward* targets, respectively. The modest positive correlation for the *Backwards* targets and the complete absence of a negative correlation for the *Forward* targets confirms that how well the target is pursued does not have a strong influence on the tapping errors. Figure 6B shows that the error in tapping is also independent of when subjects stop pursuing the target and make saccades towards the tapping area. The correlation coefficients between the tapping error and the moment at which the pursuit ended are r = 0.08 (p = 0.14), r = −0.05 (p = 0.19) and r = 0.12 (p = 0.02) for the *Backwards*, *Neutral* and *Forward* targets, respectively. The fact that the modest correlation for the *Forward* targets is positive, whereas a negative correlation would be expected if making the saccade earlier had reduced the benefit of tracking the target, confirms that pursuing the target until the tap is not essential for benefitting from tracking the target with one’s eyes.

## Discussion

When the eyes are static, local motion within a target affects how the target’s motion is perceived and, as a consequence, influences actions towards the target^34^. The results of the current study show that the effect of local motion within a moving target is very much reduced when tracking the target with one’s eyes. Thus, we can clearly conclude that there are situations in which tracking the target with one’s eyes reduces systematic interception errors that arise from misjudging the target’s retinal motion.

It is not surprising that local motion within a moving object influences its apparent speed. In order to judge the velocity of the object as a whole, the visual system has to separate motion signals arising from the object’s displacement from any other motion signals. Such other motion signals arise in daily life when an object is rolling rather than sliding, when shadows are projected onto a moving object, when parts of the object are not attached to the surface, and so on. Identifying the global motion can be fairly complicated, presumably taking place at quite a late stage of visual processing (beyond MT^47^). Any error in separating the global motion from other motion signals will lead to errors.

Souto and Kerzel^48^ have shown that the congruency between a target’s global motion and local motion signals influences how the object is pursued. In their study, targets resembled rotating wheels that were viewed from the side, whereby the rotation (local motion) was either consistent with the translation (global motion), as if the wheel were rolling, or inconsistent with the translation. Their subjects were better at tracking objects with consistent than with inconsistent motion signals. Our results are consistent with their interpretation in terms of there being a straightforward physical interpretation: we found higher pursuit gains for the *Forward* and *Neutral* stimuli, which could be considered to be rolling and sliding respectively, than for the *Backwards* stimuli in which the local and global motion had no straightforward common interpretation.

**Figure 7:**
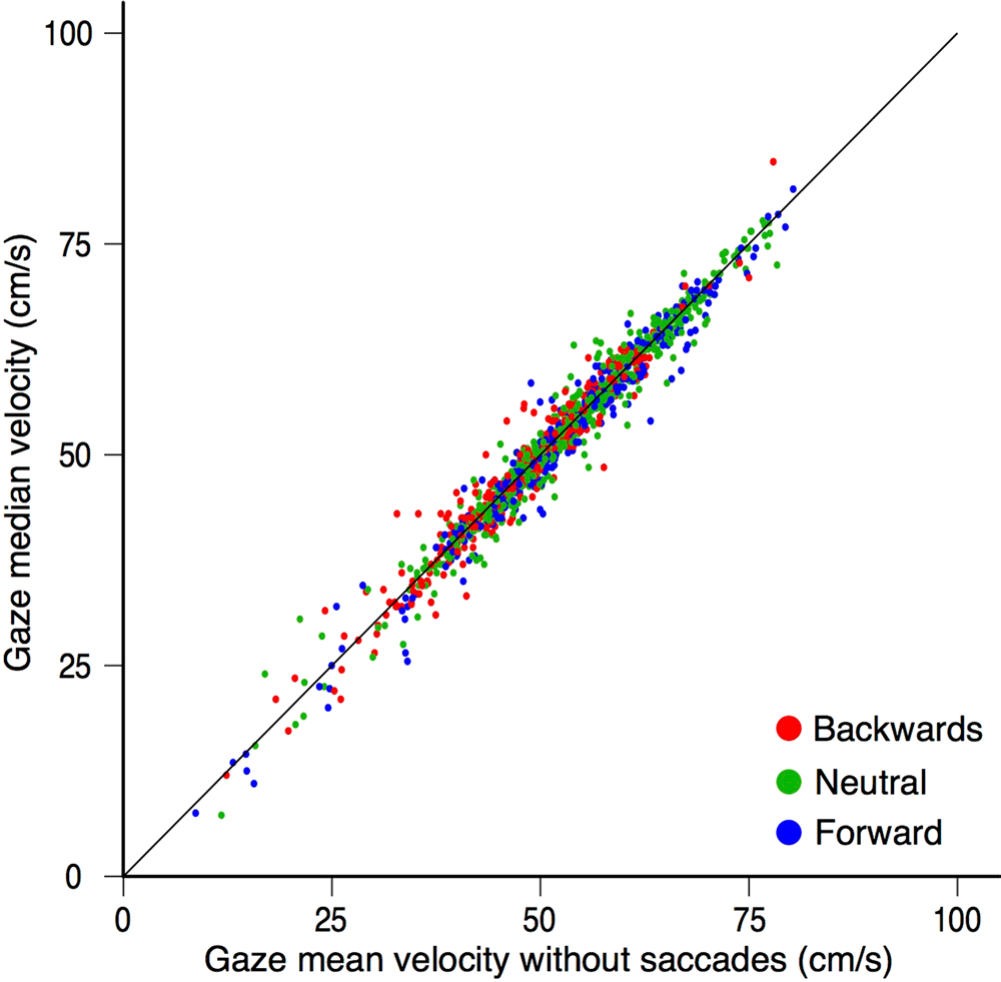
Comparison of two methods for determining the smooth pursuit component of gaze velocity: relying on the median or relying on the mean after removing saccades. Each dot represents one trial. Types of trials are colour-coded.

We rely on the median velocity as our measure of smooth pursuit. One might doubt whether this method of calculating the pursuit gain is as reliable as identifying and removing saccades from the eye movement traces before averaging the velocity. As shown in Fig. 2, in the presence of a catch up saccade, the use of the median provides a very good estimate of the smooth component of pursuit. However, this is a single example. We therefore also calculated the mean velocity after removing saccades for all trials, and compared the outcome with the median velocity that we used. Figure 7 shows that the two estimates are almost identical. Small differences do arise when the pursuit velocity is not constant. Our method has the advantage of being very simple.

Finding that targets do not have to be pursued smoothly until the tap to decrease the systematic interception errors is consistent with the finding that tracking a target with a combination of saccades and smooth pursuit is good enough for performing demanding tasks such as hitting balls in baseball, cricket and table tennis^18–20^. Such studies have shown that players do not track the targets smoothly, possibly because the balls move too fast^49^ and change direction too abruptly for players to be able to pursue them smoothly with their gaze. They make saccades to strategic places instead^19, 50, 51^. In some cases, such saccades might be essential for being able to track the target at other times.

In Experiment 2, subjects very often made saccades to where they would tap the target within the period that we expected to be the most important for their performance: between 300 and 100 ms before the moment of the tap (Figs 4 and 5). It is not clear from our study whether the fact that the target was tracked smoothly at some time before the tap was enough to reduce the influence that local motion within the target has on the judged motion of the target, or whether the target does not have to be tracked smoothly at all to obtain reliable information about the target’s movement, as long as it is tracked.

This is not the first study to suggest that it may not be necessary to pursue a target smoothly and continuously in order to benefit from making eye movements when interacting with it. Cesqui *et al*.^52^. examined whether how well people pursued a moving target correlated with their success in a catching task. The accuracy with which the target was tracked with the eyes did not influence performance, although looking at the target for a longer time did lead to more successful performance. López-Moliner and Brenner^53^ showed that in a task in which subjects had to divert their gaze to a second stimulus while trying to catch a ball that was thrown to them, the times at which they diverted their gaze were quite flexible without this affecting how successful they were at catching the ball. Thus it is clear that there is no need to pursue the target smoothly throughout its trajectory to be able to intercept it.

Another way in which we can evaluate the role of tracking the target in the present study is by comparing across target types rather than across experiments. In Experiment 1 the pursuit gain was almost the same for the *Neutral* and *Forward* targets (0.93 in both cases), and the systematic error in interception was also almost the same (0.34 and 0.35 cm for the *Neutral* and *Forward* targets, respectively). The difference in gain between the *Neutral* (0.93) and *Backwards* (0.83) targets was about 0.1, which for a target moving at about 60 cm/s for about 100 ms (the time during which error can no longer be corrected^43^) would give rise to a difference in position of about 0.6 cm. This is about half of the difference that we found (1.2 cm). Thus, the correlation between the differences between gains of smooth pursuit and the differences in tapping errors is reasonable, but not perfect. Of course, this is only a correlation, just like the modest correlation across trials for the *Backwards* targets (Fig. 6). Tracking and tapping may not influence each other, but just be influenced by the perceived speed in the same manner.

Why did subjects track the targets less well in our second experiment, in which the position at which the tracked target had to be tapped was specified? In a previous study, Brenner and Smeets^13^ found that subjects made different eye movements when they had to hit a relatively large target by moving through a small gap than when they had to hit a small target into a larger gap. In both cases subjects directed their gaze towards the smaller structure (also see ref. 54). In Experiment 2, we had to find a compromise between wanting to specify where the tracked target was to be hit and not wanting to make subjects look at that position, so we made the range within which subjects had to hit the target almost the same size as the target itself. If this range had clearly been larger than the target, subjects probably would not have made saccades towards the position of the hit just before hitting^54^, but we would not have been sure that they actually considered this range. The fact that they made these saccades and that they tapped close to the centre of this range shows that subjects did consider that they had to tap the target within the specified region. Thus, the differences that we find between the Fixation and Tracking conditions of Experiment 1 must really be due to the different eye movements, rather than to the additional constraint of having to tap the target at an indicated position in the Fixation condition.

The above-mentioned compromise is responsible for more subjects and trials being excluded in Experiment 2 than in Experiment 1: subjects often made saccades to the position at which the target would be tapped when they should have been tracking the target. Presumably, they could afford to do so because visual information earlier during the trial was enough to anticipate the target’s future trajectory^13, 16, 46, 51, 55^. Such information is updated throughout the action, leading to more accurate performance if the target’s motion is considered for a longer time^13, 56^, but at some time sensory-motor delays limit the possibility to correct the on-going action^43, 46^, so tracking the target will no longer be as beneficial. Assuming that the subjects in Experiment 2 made a saccade to the tapping area when the benefit of tracking became smaller than the benefit of checking on the tapping area, the gaze data of the Tracking condition could be interpreted as showing that tracking becomes less important about 150 ms before the tap, but the true moment may be slightly earlier than it might appear from Fig. 5 because we excluded trials and subjects when tracking stopped much earlier.

## Conclusion

Tracking a target with one’s eyes makes one much less susceptible to at least one of the many factors that are known to influence judgments of motion: local motion within a moving target. It is therefore advisable to pursue a spinning ball with one’s eyes if one wants to catch it. The eyes do not have to pursue the target particularly precisely until the moment of the tap for obtaining this benefit.
